# Consultation skills of final year medical students in Sweden: video-recorded real-patient consultations in primary health care assessed by Calgary-Cambridge Global Consultation Rating Scale, a pilot study

**DOI:** 10.15694/mep.2019.000088.2

**Published:** 2020-01-09

**Authors:** Sven-Olof Andersson, Annika Bardel, Malin André, Per Kristiansson

**Affiliations:** 1 Uppsala University

**Keywords:** Medical students, consultation skills, real patients, video-recordings, Cambridge-Calgary Global Consultation Rating Scale, patient centeredness

## Abstract

This article was migrated. The article was marked as recommended.

**Introduction:** Doctor-patient consultation is an essential element of high quality health care. Education and training of medical students in consultation skills is important. The aim of this study was to investigate the medical students’ consultation skills before graduation by assessment of the students’ video recordings of consultations with real patients at primary health care centres.

**Methods:** All students had to make a video recording of a meeting with a real patient for formative examination. 26 students participated in the study and delivered a video recording and a self-assessment. Four general practitioners assessed the video recordings by Calgary-Cambridge Global Consultation Rating Scale (CC-GCRS). Statistical testing included comparisons between groups of students and assessors using non-parametric methods.

**Results:** The average CC-GCRS-rating was higher for female students. The students’ strengths were related to relation and problem exploration. Their limitations were related to patient’s perspective, providing structure and providing information. The students assessed their consultation skills higher than the assessors did, while the relative levels were similar. The distribution of rating scores across the assessors was small.

**Conclusion:**Consultation skills were acceptable for most medical students, although there was room for improvement regarding patient centeredness skills. CC-GCRS was feasible and might be a valuable instrument to assess consultation skills for medical students at the end of their medical education.

## Introduction

Doctor-patient consultation is an essential element of high quality health care, improving patient satisfaction, recall, understanding, adherence, and outcome of care ( Kurtz *et al.*, 2003). Physicians with good communicative capability easily get comprehensive histories from the patients, more often leading to correct diagnoses and good relations with patients ( Kurtz *et al.*, 2003). In addition, important aspects of patient-centeredness which are crucial for the quality of consultation, include the concrete questioning of patients’ concerns and perspectives about their problem ( Stewart *et al.*, 2000; Larsen and Neighbour, 2014).

Education and training of medical students in consultation skills have had increased importance over the last decades. Common ideas of essential components of the education, training and curriculum, often incorporating the ideas outlined in the Calgary-Cambridge Guides, have successively been developed ( Makoul and Schofield, 1999; Makoul, 2001; Van Dalen *et al.*, 2001; Kurtz *et al.*, 2003; Silverman, Kurtz and Draper, 2013). A coherent curriculum for consultation skills training embedded in student-, patient- and community-oriented values with rehearsals every year seems to be important ( Deveugele *et al.*, 2005).

The question of how students’ communicative abilities should be assessed has had increased interest ( Duffy *et al.*, 2004). Objective Structured Clinical Examination (OSCE) with the use of Simulated Patients (SPs) giving the possibility of feed-back from both SPs and teachers have been used. However, this demands great resources and the assessment of consultation skills with the use of rating scales is under permanent discussion and development ( Comert *et al.*, 2016). The Calgary-Cambridge Global Consultation Rating Scale (CC-GCRS) is one of these scales ( Burt *et al.*, 2014). Comparisons with the students’ self-assessment may further improve the education ( Van Dalen *et al.*, 2001; Ammentorp *et al.*, 2013) as well as the use of video recordings of consultations with SPs ( Burt *et al.*, 2014). However, meeting with real patients (RP), often used in early patient contacts ( Graungaard and Andersen, 2014), seldom seems to be used for the assessment of consultation skills of medical students. The undergraduate training of medical students in consultation skills seems to positively affect their future performance ( Dong *et al.*, 2015; Gude *et al.*, 2017) and thus is crucial for the future quality of health care.

At our university, all the students during their last clinical practice at primary health care centres (PHCC) had to make a video recording of the meeting with an RP, to be shown in small groups of students with a tutor for feedback and formative examination. The recordings showed that some students experienced difficulties in the consultations possibly connected to deficiencies in education and training of consultation skills during their undergraduate studies.

## Methods

The aim of this study was to investigate the medical students’ consultation skills before medical school graduation by assessing the students’ video recordings of consultations with RP at PHCC and to compare the assessments of the assessors with the students’ self-assessments by CC-GCRS ( Kurtz *et al.*, 2003; Burt *et al.*, 2014). With this aim, a cross-sectional study was designed.

### Setting

At the medical school, Uppsala University, Sweden, all the students during their clinical practice during the final year, which was at PHCC, had to make a video recording of the meeting with an RP to be shown in small groups of students with a tutor for feedback and formative examination. During previous semesters, the students had repeatedly made video recordings of student-RP-encounters followed by assessment and feedback from physicians at the PHCC and peers. In addition, consultation skills training sessions, with SPs, occurred at semesters 1 and 8.

### Participants

During the final year the authors gave a lecture about consultation skills, according to Calgary-Cambridge Guide, including patient-doctor consultation training with role-playing ( Kurtz *et al.*, 2003). In total 160 students attended the lecture in autumn 2016 and spring 2017. At the end of each lecture, we gave the students oral and written information about the study. Fifty-nine students approved participation, out of which 26 (26/160=16%) students succeeded to deliver a video recording. Failing to deliver a recording was usually down to no patient permission and technical reasons.

### Video recordings

For a two-week period following the lecture, all medical students had clinical training, with supervision from physicians at the different PHCCs mostly in the Uppsala Region. All students performed patient encounters and video recorded at least one of them. Students participating in the study asked patients for participation after oral and written information before the video recording. The medical school provided all PHCCs with video cameras and memory cards.

### Calgary-Cambridge Global Consultation Rating Scale (CC-GCRS)

Our research group translated the CC-GCRS from English to Swedish. To validate the translation, a back-translation of the form to English was made by a person with English as their parent tongue and we found our translated version acceptable. The questionnaire held 37 items distributed in nine domains: “Initiating the session”, “Gathering information”, “Building the relationship”, “Providing structure”, “Providing information”, “Aiding accurate recall and understanding”, “Achieving a shared understanding: incorporating the patient’s perspective”, “Planning shared decision making” and “Closure”.

The domain “Gathering information” was divided into three subgroups (“Problem identification”, “Problem exploration” and “Patient’s perspective”) and “Building the relationship” into two subgroups (“Non-verbal communication” and “Developing Rapport”), totally resulting in 12 different parts.

Every item was assessed with a 9-point-scale, where 1-3 points corresponded to “Ineffective use of skill set or not done”, 4-6 points, “Reasonably competent use of skill set” and 7-9 points, “Adept or sensitive use of skill set”.

For each part of the questionnaire, an average value for the included items of all four assessors were divided by the number of items holding an assessment resulting in an average part sum score. A total average sum score was calculated by the sum of all average part sum scores divided by the number of parts holding an average part sum score.

### Video assessments

Four general practitioners separately assessed each video recording once. The assessors tried to relate the CC-GCRS scores to the expected level for a newly graduated physician. The recordings were displayed on a video screen in the same room. The assessors had already assessed students’ recordings from previous semesters as part of a pilot study. This was so they would be acquainted with CC-GCRS before the present study.

All participating students performed a self-assessment of their own consultation using the CC-GCRS.

### Ethical approval

The study was approved by the Ethical committee of Uppsala University, 2016/450.

### Statistical analyses

Summary statistics were calculated using standard methods. Standard deviation (SD) for dispersion and standard error (SE) for the means are presented. Because of the limited number of participants, non-parametric tests were used: Wilcoxon’s non-parametric test in comparison between groups, Signed Rank Test in comparison of differences between groups and Spearman’s ρ in correlations analyses. Multiple linear regression was used to analyse associations between dependent and independent variables adjusting for possible confounding factors. A p-value <0.05 was considered statistically significant. Statistical analysis was performed using the SAS program package version 9.4 (SAS Institute, Cary, NC, USA).

## Results/Analysis

Twenty-six medical students (16 females and 10 males) in the final year of the medical school completed a student-patient video-consultation. The mean age of the students was 26 years (S.D. 2.8) with a range between 20 and 33 with no age difference between the sexes.

### General Practitioners’ assessment

The total average sum score for all students was 5.0 points (SE 0.16), for female students 5.3 (SE 0.19) and for male students 4.6 (SE 0.29) (p=0.04).

The average part sum score for all students´ video consultations are shown in Table 1. The two CC-GCRS parts with the highest average part sum scores were “non-verbal communication” and “initiating the session”. The two CC-GCRS parts with the lowest average part sum scores were “patient’s perspective” and “incorporating the patient’s perspective”.

**Table 1.** The sum score of Calgary-Cambridge Global Consultation Rating Scale for medical students’ video consultations assessed by GPs ^*)^ and the students’ self assessments.

CC-GCRS ^**)^ score	GPs' assessments		Students' assessments		Differences between GPs´ and students´ assessments		
	n	Mean (sd)	n	Mean (sd)	n	Mean (sd)	p-value ^***)^
All twelve parts	26	5.0 (0.8)	25	6.7 (1.0)	22	1.5 (0.9)	<0.0001
Initiating the session	26	5.4 (1.0)	25	7.2 (1.0)	22	1.5 (1.1)	<0.0001
Problem identification	26	5.1 (1.0)	25	6.9 (1.0)	22	1.6 (1.1)	<0.0001
Problem exploration	26	5.3 (0.9)	25	7.1 (1.1)	22	1.5 (1.0)	<0.0001
Patient’s perspective	26	4.4 (1.5)	24	6.1 (1.8)	21	1.7 (1.6)	<0.0001
Non-verbal communication	25	5.7 (0.9)	25	7.3 (1.1)	22	1.4 (1.0)	<0.0001
Developing rapport	26	5.2 (1.0)	25	7.2 (0.9)	22	1.7 (0.9)	<0.0001
Providing structure	26	4.8 (0.8)	24	5.8 (1.7)	21	0.8 (1.8)	0.03
Providing information	23	4.8 (1.0)	22	6.4 (1.3)	16	1.8 (1.5)	0.0004
Aiding in accurate recall and understanding	22	5.1 (1.0)	24	6.2 (1.4)	17	1.2 (1.6)	0.003
Incorporating the patient’s perspective	21	4.4 (1.3)	23	6.2 (1.3)	16	1.9 (1.2)	<0.0001
Planning and shared decision making	16	4.9 (1.3)	22	6.4 (1.9)	12	1.5 (2.2)	0.05
Closure	13	5.3 (1.2)	21	7.4 (1.2)	9	1.9 (1.6)	0.008

^*)^ General Practitioner

^**)^ Cambridge-Calgary Global Consultation Rating Scale

^***)^ p-value (Signed rank test) refers to the assumption of no difference between the assessment groups.

The female students got higher average part sum scores compared with male students among the CC-GCRS parts: initiating the session (5.8 vs 4.8, p=0.008), problem identification (5.8 vs 4.7, p=0.009) and problem exploration (5.7 vs 4.6, p=0.005). For the other average part sum scores there were no significant differences between the sexes.

In a correlation analysis, there were positive correlations between the average part sum scores (0.45≤Spearman’s ρ ≤0.96, <0.0001≤p≤0.045), except among 11 of the 144 combinations (data not shown). This means that students with a high score in one CC-GCRS part also had high average part sum scores in most other CC-GCRS parts. For the CC-GCRS part “patient’s perspective” the average part sum score varied between 1.8 and 7.1, where eleven students (42%) were assessed with a score <4 (“Ineffective use of skill set”) and one student (3.8%) was assessed with a score ≥7 (“Adept or sensitive use of skill set”).

**Figure 1.**
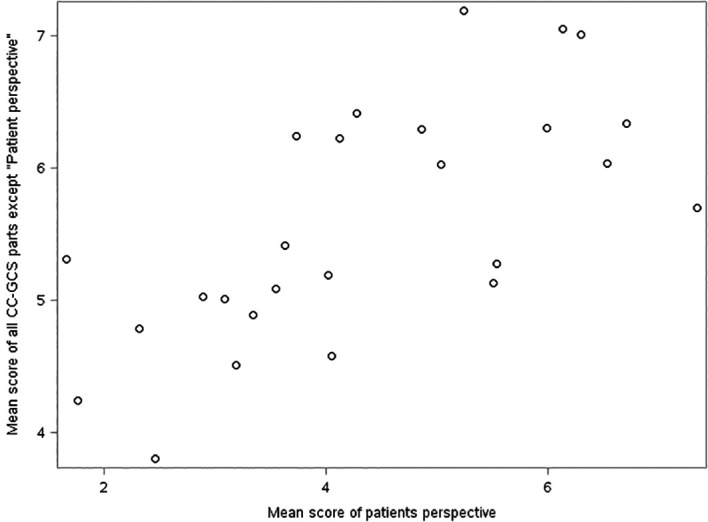
Assessment of 26 medical students’ student-patient video consultations by Cambridge-Calgary Global Consultation Rating Scale (CC-GCRS) by general practitioners.

In simple linear regression analyses, there was a positive association between the average part sum score of “patient’s perspective” and the total average sum score of all other CC-GCRS parts (β=0.31, p=0.002) with an R ^2^ of 0.32, Figure 1. In a multiple linear regression analysis, with the same dependent and independent variables, adjusted for sex of the student, the part “patient’s perspective” (β = 0.28, p=0.004) and sex were independently associated (β = 0.59, p=0.04) with the total average sum score of all other CC-GCRS parts. R ^2^ of the full model was 0.44 (p=0.002).

### Student’s self-assessment

The total average sum score assessed by each student was 6.7 (SE 0.20), Table 1.

The two CC-GCRS parts with the highest average part sum score were “Closure” (7.4, SE 0.26) and “Non-verbal communication” (7.3, SE 0.23). The two CC-GCRS parts with the lowest average part sum score were “Patient’s perspective” (6.1, SE 0.37) and “Providing structure” (5.8, SE 0.58).

### Comparison between assessments by assessors and students

There was a positive correlation between the total average sum scores between the assessments by the assessors and the student (Spearman’s ρ =0.51, p=0.02). The total average sum score of the students’ assessments was 1.5 (SE 0.2) points higher, than that of the assessors (p<0.0001), Table 1. The difference between each of the CC-GCRS total average sum score, assessed by the assessors and students, ranged between 0.8 (SE 0.38) and 1.9 (SE 0.52), (<0.0001<p≤0.05). The parts of CC-GCRS with greatest differences between the assessments by the assessors and the students were “Patient’s perspective”, “Developing rapport”, “Providing information”, “Incorporating the patient’s perspective” and “Closure”.

### Comparisons between assessors

The total average sum score for each of the four assessors was 4.7, 4.8, 5.2 and 5.5. The least significant difference between the assessors’ assessments was 0.56 which explained 8% of the variation of the total average sum score (p=0.04).

## Discussion

Final year medical students’ consultation skills were in total assessed as “reasonably competent use of skill set”, in this cross-sectional study. The highest skills comprised abilities that do not demand specific consultation skills. However, the important aspects, patient perspectives and providing structure, showed room for improvement. The rating was slightly higher for females than for males and the distribution of rating scores across the assessors was small. In addition, the students assessed their consultation skills higher than the assessors did, while the relative levels were similar. The experiences from the present study could be used in future improvement of the education of consultation skills.

We noted that the students got the highest score concerning the CC-GCRS-domains “initiating the consultation session” and “non-verbal communication”, which are performances that do not demand specific consultation skills. However, a considerable proportion of the students were assessed as “ineffective use of skill set” and a very small proportion were assessed as “adept or sensitive use of skill set” regarding the important item “patients’ perspective” including active exploration of patients’ ideas, concerns and expectations. Our results also indicated that students good at clarifying “patients’ perspective” were also good at almost all other parts or domains of the consultations. This could imply that actively determining the “patients’ perspective” was crucial for the quality of the medical students’ consultations.

The female students in the present study had higher total CC-GCRS score and the CC-GCRS domains “initiating the session”, “problem identification” and “problem exploration” is in accordance with previous studies ( Graf *et al.,* 2017; Sugawara *et al*., 2017). The suggestion of gender-specific teaching might be taken under consideration.

The total average sum score for all students had quite small standard deviations. This might mean that this rating scale would work well for formative and summative evaluation of learning and training of consultation skills. In addition, since the dispersion for the four different assessors was small, the assessment of fewer examiners could be acceptable. This is in accordance with high reliability scores presented by Burt *et al*. for two or three raters ( Burt *et al.*, 2014).

Students’ self-evaluation can be useful for measuring relative performance of student’s consultation skills ( Weiss *et al.*, 2005; Wall, Bolshaw and Carolan, 2006; Gude *et al.*, 2017). However, the absolute scores should be interpreted with caution as has been shown in comparisons of patients’ assessment and trained clinical raters ( Burt *et al.*, 2018). The students will have the possibility to find their specific strengths and limitations by using CC-GCRS and train consultations skills accordingly. In addition, we believe that the actual use of the CC-GCRS gives a valuable effect on students’ understanding of the consultation structure. Furthermore, the fact that the students rated themselves higher than the assessors did might depend on how the students used the questionnaire in relation to their actual consultation. They may have filled-in the questionnaire without scrutinizing the meaning of the questions in relation to the video-recording but from their memory of the consultation.

RPs are crucial in clinical education and training especially in the clinical investigation of patients and training in clinical reasoning etc. ( Spencer *et al.*, 2000). However, RPs are seldom used to assess the consultation skills of medical students. Instead, simulated or standardized patients are mostly used. This makes comparisons of our results hard ( Gallagher, Hartung and Gregory, 2001; Yedidia *et al.*, 2003; Bokken *et al.*, 2009; Schaufelberger *et al.*, 2012). In our study, the students could get any patient to interview and record, and often had to recruit the patients themselves or together with the staff at the PHCC. The situation for the student is therefore different from interviews with SP and in OSCEs. Despite these difficulties, our motivation to use RPs in the study of the students’ consultation skills was to get an idea of the students´ preparedness for their work as physicians after graduation.

### Strengths and limitations

CC-GCRS has high reliability and validity ( Burt *et al.*, 2014) and is a widely used and spread instrument based on the Cambridge-Calgary Guide, grounded on a solid scientific base ( Silverman, Kurtz and Draper, 2013). The use of RPs in the consultation setting was a strength as was the number of raters. The motivation for the use of student self-assessment to understand the consultation, was to teach the students the structure and specific content of good consultations. However, whether this really worked or not was not confirmed in our study.

There were several limitations in our study. Due to the low participation rate the generalizability of the study results to the whole medical student population is weak. Thus, the results must be interpreted with caution and be confirmed in a future study. Explanations to the low numbers of students delivering a student-patient video recording were difficulties in recruiting patients, technical video recording problems and the time of the study at the very end of the final year and forthcoming graduation. One technical problem was that only half of the students succeeded to record a completed consultation. This resulted in fewer assessments of the closure of the consultation. This means that further studies need more resources for administration.

## Conclusion

Consultation skills were acceptable for most medical students at their final year in medical school, although regarding patient centeredness skills there was room for improvement. In the education of medical students, “patient perspective” including the active exploration of patients’ ideas, concerns, expectations, feelings and effects on life must be further highlighted. This might be generally true for the curriculum and the training sessions at the medical schools from the beginning to the end of the education.

## Take Home Messages

• The average CC-GCRS-rating was higher for female students.
• The students’ strengths related to relation and problem exploration and their limitations were patient’s perspective, providing structure and providing information.
• The students assessed their consultation skills higher than the assessors did, while the relative levels were similar.
• CC-GCRS was feasible and might be a valuable instrument to assess consultation skills.

## Notes On Contributors

Sven-Olof Andersson, M.D., Ph.D., is a general practitioner and has been teaching in medical psychology and professional development for many years. His main interests include consultation and communication in general practice.

Annika Bardel, M.D., Ph.D., is a general practitioner and adjunct lecturer in general practice. Her main interests include education of tutors for medical students in general practice.

Malin André, M.D., Ph.D., is a general practitioner and researcher in general practice for many years.

Per Kristiansson, M.D., Ph.D., is associate professor and head of the unit of general practice, Uppsala University. A main interest include consultation and communication in general practice.
