# Supplementary material for: Consultation skills of final year medical students in Sweden: video-recorded real-patient consultations in primary health care assessed by Calgary-Cambridge Global Consultation Rating Scale, a pilot study
Source: MedEdPublish (2016). 2020 Jan 9;8:88. Originally published 2019 Apr 16. [Version 2] doi: 10.15694/mep.2019.000088.2 (PMC10712619; doi:10.15694/mep.2019.000088.2)
Supplement: Peer review history

# Reviewer report: Reviewer response for version 2

**DOI:** 10.21956/mep.20245.r31394

**Published:** 2020-04-02

**Authors:** Ken Masters

**Affiliations:** 1 Sultan Qaboos University

This review has been migrated. The reviewer awarded 4 stars out of 5 A small and localised study, but with encouraging results that could lead to larger work, and be of value to other medical educators. The authors have done well to address the concerns of the previous reviewers. An interesting follow-up research paper (in 5 years’ time) would be to have those same students, now doctors, re-evaluate their own videos. Their grades and their comments, based on 5 years’ experience, would be interesting.

Reviewer Expertise:

NA

No decision status is available

# Reviewer report: Reviewer response for version 2

**DOI:** 10.21956/mep.20245.r31395

**Published:** 2020-01-16

**Authors:** Balakrishnan Nair

**Affiliations:** 1 Centre for Medical Professional Development and University of Newcastle

This review has been migrated. The reviewer awarded 4 stars out of 5 Connecting with patients and providing patient centred care is the core of medical practice. The curriculum should prepare graduates to become good doctors rather than just providing knowledge. So this study looking at the consultation skills of final year students should be commended. However they faculty taught 160 students , 59 consented and only 26 videos were available for analysis. In the discussion , this selection bias and Hawthorn effect should have been discussed. Overall a well done study

Reviewer Expertise:

NA

No decision status is available

# Reviewer report: Reviewer response for version 2

**DOI:** 10.21956/mep.20245.r31396

**Published:** 2020-01-09

**Authors:** Felix Silwimba

**Affiliations:** 1 University of Lusaka

This review has been migrated. The reviewer awarded 5 stars out of 5 this is a good study and informative. Although, I'm confused in applying deductive and inductive approaches to research.I would have thought such a study could have more qualitative than using quantitative methods in the interpretation of results. You can help appreciate the statistics used in this study compared to a grounded theory approach.

Reviewer Expertise:

NA

No decision status is available

# Reviewer report: Reviewer response for version 1

**DOI:** 10.21956/mep.19123.r27603

**Published:** 2019-06-30

**Authors:** Debra Kiegaldie

**Affiliations:** 1 Holmesglen Institute & Healthscope Australia

This review has been migrated. The reviewer awarded 3 stars out of 5 A very interesting small scale study that would benefit from a good editorial ‘eye’ to address some for phrasing and syntax errors. This would tighten the paper considerably. Is there another way of saying ‘last semester’ – this could be misconstrued as meaning any preceding semester – Perhaps final year? Pre-graduation? The instruments used in the study are appropriate and well analysed. The results section is written very well. I would be interested in the authors exploring more about the gender difference.A few other things to note: I prefer the term simulated patient rather than standardized or even simulated participant. I feel we are moving away from that terminology now. Rather than referring to Uppsala, perhaps state, at our university. Methods should start with: The aims of this study were to…..Overall a worthy paper.

Reviewer Expertise:

NA

No decision status is available

# Reviewer report: Reviewer response for version 1

**DOI:** 10.21956/mep.19123.r27604

**Published:** 2019-04-17

**Authors:** BALAJI ARUMUGAM

**Affiliations:** 1 TAGORE MEDICAL COLLEGE AND HOSPITAL

This review has been migrated. The reviewer awarded 4 stars out of 5 Consultation skills of medical students – a very useful topic well presented and analyzed in all the dimensions using a 37 item questionnaire focusing on nine domains from initiating the session to closure of the consultation session. This study has analyzed the scores based on self assessment, assessment between the students and assessors and comparison among the assessors. The study showed great statistical association findings of which I felt the important was the gender difference that is the female students got higher average part sum scores compared with male students among the CC-GCRS parts: initiating the session, problem identification and problem exploration. Thanks and congrats to all the authors for presenting such a nice article with the standardized questionnaire.

Reviewer Expertise:

NA

No decision status is available

# Reviewer report: Reviewer response for version 1

**DOI:** 10.21956/mep.19123.r27605

**Published:** 2019-04-16

**Authors:** Felix Silwimba

**Affiliations:** 1 University of Lusaka

This review has been migrated. The reviewer awarded 5 stars out of 5 This is a good study documenting the use a standard method of student patient consultation assessment. The CC-GCRS, its good that it was applied across language and cultural differences. That is English and Swedish. It also made mention of the standardized patients and Real patients in student assessments. I find this method applicable everywhere else in the world. I recommend such studies to be replicated in low-income countries.

Reviewer Expertise:

NA

No decision status is available
